# Gender differences in psychiatric comorbidity and personality characteristics among adults seeking treatment for problematic internet use

**DOI:** 10.3389/fpsyt.2022.1022749

**Published:** 2022-10-28

**Authors:** Rodrigo Menezes Machado, Hyoun S. Kim, Vinicius Oliveira de Andrade, Lindsey A. Snaychuk, Carla Cavalheiro Moura, Cornelia Belliero Martini, Cristiano Ricardo Faedo Nabuco de Abreu, David C. Hodgins, Hermano Tavares

**Affiliations:** ^1^Faculty of Medicine, University of São Paulo, São Paulo, Brazil; ^2^Department of Psychology, Toronto Metropolitan University, Toronto, ON, Canada; ^3^Department of Psychology, University of Calgary, Calgary, AB, Canada

**Keywords:** problematic internet use, internet addiction, behavioral addictions, gender differences, psychiatric comorbidity

## Abstract

In the present study, we investigated gender differences in personality and psychiatric correlates among adults (*N* = 115) seeking treatment for problematic internet use (PIU) at a specialized clinic in São Paulo, Brazil. All participants were assessed at the beginning of their treatment for co-occurring psychiatric conditions, other addictive behaviors, and personality characteristics. Women (*n* = 20) were more likely to present with greater rates of psychiatric comorbidity compared to men (*n* = *95*), including mood disorders, anxiety disorders, obsessive-compulsive disorder, post-traumatic stress disorder, and bulimia nervosa. Women also had a greater severity of certain behavioral addictions, such as compulsive buying and disordered eating. Gender differences were also found across personality characteristics, with women scoring higher on impulsivity, novelty seeking, and self-transcendence compared to men. To our knowledge, the present study is the first to investigate gender differences for PIU in a clinical sample. The results suggest that there are notable gender differences in individuals seeking treatment for PIU which underscores the importance of assessing for co-occurring conditions, especially in women. Understanding the characteristics associated with PIU can help serve to inform the most appropriate interventions to bolster treatment outcomes.

## Introduction

The internet, as we know it today, started in the early 1980s and has since become a worldwide phenomenon (1–3). Although the internet presents numerous uses and benefits, some people develop a problematic pattern of use that results in varying degrees of functional impairment, such as worsening academic performance, occupational losses, and lower quality of life (4). Historically, the term “Internet addiction disorder” appeared in the mid-1990s to describe a maladaptive pattern of internet use that shared characteristics present in behavioral addictions and substance use disorders (SUDs) (3, 5, 6). Despite advances in the conceptualization of this term, there is still no consensus regarding its diagnostic criteria, and internet addiction is not included in the Diagnostic and Statistical Manual of Mental Disorders (DSM-5) or the International Classification of Diseases (ICD-11). However, problematic internet use (PIU) can be classified in two ways in the ICD-11: The first is 6C5Y-Other specified disorders due to addictive behaviors or 6C5Z-Disorders due to addictive behaviors, unspecified. Divergences are also present when defining a nomenclature, and the terms “compulsive internet use,” “problematic internet use (PIU)” and “internet addiction” have been used in the literature (7, 8). The term PIU (which we adopt in the present study) was designed to create a distinction in the classification of addiction until scientific evidence accumulated and there was a greater understanding of the disorder (9, 10).

Problematic internet use is characterized by “excessive or poorly controlled preoccupations, urges, or behaviors regarding computer use and internet access that leads to impairment or distress” (11). The Internet Addiction Test (IAT) developed by Young (4, 12) is one of the most frequently used instruments to identify PIU and has been shown to have strong psychometric properties (13). Based on the DSM-IV definitions of SUD and gambling disorder (GD), Young proposed the following criteria for “internet addiction”: withdrawal, tolerance, preoccupation with being online again, more time than intended spent on the internet, significant risks in relationships and/or occupation, lying about internet use, and repeated and unsuccessful attempts to cut down and/or quit internet use (3, 12). A recent meta-analysis conducted by Pan et al. (14) found a prevalence rate of 7.02% (95% CI, 6.09–8.08%) in the general population suggesting that PIU is relatively common. Unfortunately, PIU is associated with negative impact on everyday functions, family relationships, and emotional stability (15, 16). In addition, PIU may be positively associated with incarceration, legal problems, and physical and mental impairments (17–19). Given the conceptualization of PIU as a behavioral addiction, it is not surprising that PIU frequently co-occurs with other psychiatric disorders, in line with previous findings of the high rates of comorbidity in gambling (18, 20). For example, previous research has found that PIU frequently co-occurs with anxiety disorders, mood disorders, addictions, and personality disorders (21–24) with almost 50% of individuals with PIU meeting criteria for another co-occurring disorder (22, 24).

Gender differences have also been found in PIU. For example, men are relatively more likely to develop generalized PIU than women, particularly in Asia, with less pronounced differences in Europe, North America, and Africa (25). Additionally, women tend to have a predilection for using social networking and online shopping, while men are more likely to use the internet for online gaming, gambling, and pornography (26, 27). In addition, although previous studies suggest that men tend to present with more severe PIU symptoms (28, 29), other studies do not find these gender differences (30) or have found that women reported greater symptoms of PIU severity (31). Gender differences have also been found in the pathways to PIU as well as in patterns of comorbidities (31, 32). In a large study of adolescents (*n* = 2,114), greater severity of PIU symptoms was associated with higher rates of ADHD and depression between the genders, however, higher rates of aggression were associated with increased PIU severity but only among men (33). With respect to social aspects, there seems to be a significant association between social anxiety disorder (SAD) and PIU, which is stronger for men than for women (34). Interestingly, in a large study of adolescents (35), depression significantly predicted the development of subsequent PIU in men only, suggesting that depression may be a causal factor of PIU in this population, perhaps as a maladaptive coping mechanism. Whereas, in female adolescents, PIU significantly predicted subsequent depression, suggesting important gender differences in PIU.

In summary, notable differences have been found between men and women with PIU. Unfortunately, most studies addressing PIU have used non-clinical populations with a considerably smaller number of investigations of clinical samples. Similarly, most studies investigating gender differences in PIU have done so in non-clinical samples. Indeed, to our knowledge, no study to date has examined gender differences in PIU among people who are seeking treatment for PIU. The lack of studies using clinical samples provides an incomplete understanding of PIU, given that people who seek treatment are different from those who do not (36). Given the existing literature gap addressing PIU with clinical samples, this study aimed to verify the potential associations between demographic variables, addictive behaviors and other psychiatric comorbidities, and gender differences in PIU patients who voluntarily sought treatment at a specialized healthcare service in São Paulo, Brazil.

## Materials and methods

### Participants and procedures

The study sample consisted of 115 individuals who voluntarily sought treatment for PIU at a specialized healthcare service in São Paulo, Brazil. Twenty participants self-identified as a woman and 95 self-identified as a man. This healthcare service is located at a university hospital that delivers treatment free of charge and is the largest specialized treatment center for impulse control disorders in Brazil. Patients can self-refer or be referred from other services within the hospital to seek treatment. All study participants were assessed at the beginning of their treatment. During the screening process, patients completed a Brazilian Portuguese version of the IAT (3, 13, 37). Patients whose scores indicated possible presence of PIU were assessed with an adapted version of the Structured Clinical Interview for DSM (ICD-SCID) with a registered psychiatrist to diagnose the presence of PIU. Following the treatment center protocol, the diagnosis of PIU was then confirmed by a licensed psychologist, who also collected additional clinical measures. The clinical measures included those relevant to the present research (addictive behaviors, personality) as well as other measures including social impairments, mental health (e.g., depression, anxiety, attention deficit, and binge eating) and measures of hostility and sexual impairment. A semi-structured (detailed below) was used to diagnosis mental health disorders.

The treatment protocol was adapted from Kimberly Young’s Cognitive Behavioral Therapy – Internet Addiction (CBT-IA) (38) for use in Brazil (39). The CBT-IA was delivered in group format and consisted of 18 sessions and consisted of three treatment phases as per CBT-IA: Phase I: Behavior Modification, Phase II: Cognitive Restructuring, and Phase III: Harm Reduction. Additionally, some patients are provided with pharmacological treatments to treat co-occurring psychological disorders (e.g., depression and anxiety) when clinically appropriate.

All eligible patients were informed about participation in the research study and signed an informed consent to participate in the research. This study was approved by the Faculty of Medicine, University of São Paulo, Research Ethics Board (#12820813.5.0000.0068) and Toronto Metropolitan Universities Research Ethics Board (#2020-417).

### Measures

#### Demographic information

A sociodemographic questionnaire used at the specialized healthcare center was used to collect standard demographic information, such as age, gender, ethnicity, and marital status. We also assessed for type of internet use behaviors that the patients rated as being problematic.

#### Addictive behaviors

The Brazilian Portuguese version of the Shorter PROMIS Questionnaire (SPQ) (38) was used to investigate 16 domains of addictive behaviors: use of alcohol, tobacco, recreational drugs, prescription drugs, gambling, sex, caffeine, food binging, food starving, exercise, shopping, work, relationships – dominant and submissive, and compulsive helping – dominant and submissive. In the present research we removed the relationship and compulsive helping subscales due to their lack of conceptual similarities to addictions. Each domain consists of 10 items anchored from 0 (not like me) to 5 (like me). Each subscale is summed into a continuous score from a minimum of 0 to a maximum of 50, with higher scores indicating greater severity of the related addictive behavior problem. Items assess behaviors and attitudes related to addictive behaviors and included: “I have often gone shopping to calm my nerves” and “I have tended to think of food not so much as a satisfier of hunger but as a reward for all the stress I endure.” The SPQ validation study indicated that the alpha coefficients were acceptable with a mean alpha of 0.89 (40). The alpha from the present sample on the PROMIS ranged from 0.75 to 0.97.

#### Psychiatric comorbidities

Comorbid psychiatric disorders were assessed using the Mini-International Neuropsychiatric Interview (MINI) version validated for Brazilian Portuguese (41). The MINI is a brief, structured psychiatric interview that aims to evaluate mood and anxiety disorders, obsessive-compulsive disorder, post-traumatic stress disorder, psychosis, and SUDs. The MINI has shown strong psychometric properties when compared with those of other structured clinical interviews, such as the SCID. The MINI was validated for use in Brazil in primary centers, with kappa coefficients ranging between 0.65 and 0.85, sensitivity ranging from between 0.75 and 0.92, and specificity between 0.90 and 0.99 (41, 42).

#### Personality

The Brazilian Portuguese version of the Temperament and Character Inventory (TCI) (43) assessed seven dimensions of personality traits: four temperaments (reward dependence, novelty seeking, persistence, and harm avoidance) and three characters (cooperativeness, self-transcendence, and self-directedness). The Brazilian Portuguese version of the TCI has internal consistency similar to its original version, with Cronbach’s alpha coefficients for all dimensions above 0.70 (44, 45). Example items on the TCI include: “I usually am confident that everything will go well, even in situations that worry most people” and “I often feel a strong spiritual or emotional connection with all the people around me.” The TCI has been widely applied to assess the personality of individuals with SUDs (46) and behavioral addictions (47). In the present study, the alpha coefficients for the TCI ranged from 0.74 to 0.90.

The Barratt Impulsiveness Scale – 11 adapted for Brazilian Portuguese (BIS-11) (48, 49) is composed of 30 items that measure different aspects of impulsivity (attentional, motor, and unplanned). It is anchored from 0 (rarely/never) to 4 (almost always/always), with higher scores indicating higher impulsivity levels. Example items on the BIS-11 include: “I plan tasks carefully” and “I buy things on impulse.” The Brazilian Portuguese version of the TCI showed high correlation with the English version (*r* = 0.91–0.93) when tested on a bilingual sample of Brazilians in a community population with an alpha of 0.80 and test-retest reliability at 7 months of *r* = 0.72 (46). The Brazilian Portuguese version has been validated in several samples [e.g., adolescents (50)], and there is normative data for the general Brazilian population (51). In the present sample, the alphas ranged from 0.63 to 0.73.

### Data analytic plan

First, we examined whether our continuous variables were normally distributed using the Kolmogorov–Smirnov test. Independent samples *t*-tests were used for normally distributed continuous variables and Mann–Whitney *U* tests were used for continuous variables not normally distributed. Chi-square tests were used for categorical variables. Fisher’s exact tests were used when expected cell counts were less than five. For effect sizes, we calculated Cohen’s *d* for *t*-tests, *r* for Mann–Whitney *U* tests and Cramer’s V for Chi-square tests and Fisher’s exact tests. Benjamini–Hochberg False Discovery Rate was applied to correct for multiple comparisons. Not all participants completed all the measures of interest. Little’s MCAR test indicated that the data was missing completely at random χ^2^ (571) = 0.507.62, *p* = 0.973, and thus list-wise deletion was used for missing data.

## Results

### Demographics

A Mann–Whitney *U* test did not reveal significant differences in the median ages between women (29.0) and men (25.0), *U* = 942.0, *p* = 0.486, *r* = −0.07. Similarly, there were no significant differences between women and men regarding ethnicity with 77.3% of women and 75.5% of men self-reporting being White, χ^2^ (1) = 0.04, *p* = 0.848, Cramer’s V = 0.02, or in marital status with 83.3% of women and 80.9% of men reporting being single, χ^2^ (1) = 0.002, *p* = 0.965, Cramer’s V = 0.004. Men (67.5%) were significantly more likely to report gaming as a problematic form of internet use compared to women (38.5%), χ^2^ (1) = 4.07, *p* = 0.044, Cramer’s V = 0.21. No other differences were found in regard to problematic forms of internet use: (i) information and search pages (42.5% men, 46.2% women), χ^2^ (1) = 0.06, *p* = 0.805, Cramer’s V = 0.03, (ii) email (16.3% men, 23.1% women), Fisher’s exact test *p* = 0.691, Cramer’s V = 0.06, and (iii) chat rooms/internet messaging services (33.8% men, 30.8% women), Fisher’s exact test *p* = 1.00, Cramer’s V = 0.02.

### Addictive behaviors

The results of the Mann–Whitney *U* indicated that women had significantly higher median scores on shopping, food binging and food starving compared to men, which remained significant when correcting for multiple comparisons with moderate effect sizes. No differences in median scores were found between women and men in alcohol, tobacco, caffeine, recreational drugs, prescription drugs, gambling, sex, work, or exercise, *p*s > 0.093 (Table 1).

**TABLE 1:** Comparison of addictive behaviors as measured by the Shorter PROMIS Questionnaire between women and men.

Addictive behaviors	Men (*n* = 72)		Women (*n* = 17)		*U* statistic	*P*	*r*
	Mean (SD)	Median	Mean (SD)	Median			
Alcohol	7.53 (8.75)	5.00	10.31 (13.76)	5.50	585.00	0.634	−0.05
Tobacco	3.67 (9.92)	0.00	6.31 (14.92)	0.00	577.50	0.797	−0.03
Caffeine	5.72 (7.18)	3.00	12.94 (17.59)	3.50	618.00	0.572	−0.06
Recreational drugs	4.99 (9.10)	0.00	4.76 (12.09)	0.00	573.00	0.647	−0.05
Prescription drugs	4.38 (8.25)	1.00	13.00 (15.73)	4.00	682.00	0.093	−0.18
Gambling	10.72 (11.78)	7.00	9.59 (14.18)	2.00	550.00	0.511	−0.07
Sex	8.82 (9.94)	5.00	8.12 (13.46)	2.00	479.50	0.187	−0.14
Work	13.70 (9.28)	12.00	20.44 (15.35)	18.50	714.50	0.108	−0.17
Exercise	10.71 (7.77)	9.00	12.88 (12.25)	11.50	587.00	0.764	−0.03
Shopping	11.25 (10.67)	7.00	25.06 (14.15)	28.50	893.00	**<0.001** *	**−0.38**
Food binging	13.57 (11.03)	10.00	28.12 (15.29)	29.00	930.00	**<0.001** *	**−0.38**
Food starving	6.69 (6.71)	5.00	17.29 (12.54)	18.00	934.00	**<0.001** *	**−0.39**

Bold denotes significant differences. *Denotes significance when controlling for multiple comparisons.

### Psychiatric comorbidities

Women were significantly more likely to be diagnosed with current psychiatric comorbidities (Table 2). Specifically, women had higher rates of major depression, suicidality, social phobia, obsessive compulsive disorder, panic disorder, post-traumatic stress disorder, and bulimia nervosa. These results remained significant when correcting for multiple comparisons with moderate effect sizes. In contrast, no significant differences were found between genders regarding current diagnoses of agoraphobia, generalized anxiety disorder, alcohol dependence, or substance dependence *p*s > 0.141. No participant met diagnostic criteria for anorexia nervosa.

**TABLE 2:** Comparison of current psychiatric comorbidities between women and men.

Psychiatric comorbidity	Men (*n* = 81)		Women (*n* = 13)		χ ^2^	*P*	Cramer’s V
	*n*	%	*n*	%			
Major depressive disorder	35	43.2	11	84.6	7.69	**0.006** *	**0.29**
Suicidality	25	30.9	9	69.2	7.14	**0.008** *	**0.28**
Social anxiety	14	17.3	6	46.2		**0.029** * ^∧^	**0.24**
Agoraphobia	15	18.5	5	38.5		0.141^∧^	0.17
OCD	12	14.8	6	46.2		**0.016** * ^∧^	**0.28**
Panic disorder	2	2.5	3	23.1		**0.018** * ^∧^	**0.32**
PTSD	0	0	3	23.1		**0.002** * ^∧^	**0.45**
GAD	30	37.0	6	46.2		0.552*^∧^*	0.07
Alcohol dependence	4	4.9	2	15.4		0.192*^∧^*	0.15
Substance dependence	7	8.6	1	7.7		1.00*^∧^*	0.01
Bulimia nervosa	0	0	2	15.4		**0.018** * ^∧^	**0.37**

OCD, obsessive compulsive disorder; PTSD, post-traumatic stress disorder; GAD, generalized anxiety disorder. Bold denotes significant differences. ^∧^Fisher’s exact test was used as expected cell counts were less than 5. *Denotes significance when controlling for multiple comparisons.

### Personality

Women reported significantly higher scores on the BIS-motor, BIS total scores, novelty-seeking, and self-transcendence compared to men (Table 3). However, only BIS-motor and self-transcendence remained significant when correcting for multiple comparisons with moderate effect sizes. No differences were found regarding BIS-attention, BIS-non-planning, cooperativeness, self-directedness, reward dependence, persistence, or harm avoidance, *p*s > 0.065.

**TABLE 3:** Comparison of personality characteristics between women and men.

Personality variables	Men (*n* = 72)		Women (*n* = 17)		Test statistics	*P*	Effect size
	Mean (SD)	Median	Mean (SD)	Median			
BIS – attention	20.06 (4.46)	19.20	22.21 (4.14)	20.80	747.00^a^	0.079	−0.19^c^
BIS – motor	20.46 (4.95)	20.17	25.21 (6.75)	22.61	851.00^a^	**0.003** *	**−0.32^c^**
BIS – non-planning	28.54 (5.60)	–	29.63 (5.64)	–	−0.70^b^	0.484	0.20^d^
BIS – total	69.56 (12.79)	68.75	76.88 (13.02)	73.13	711.00^a^	0.032	−0.23^c^
Cooperativeness	27.21 (6.35)	27.50	24.19 (6.22)	25.00	397.50^a^	0.095	−0.18^c^
Self-directedness	19.73 (7.29)	20.50	15.67 (7.21)	15.00	341.00^a^	0.061	−0.21^c^
Reward dependence	12.86 (4.42)	–	14.18 (4.33)	–	−1.09^b^	0.276	0.30^d^
Persistence	3.56 (2.01)	3.00	3.88 (1.90)	4.00	653.00^a^	0.530	−0.07^c^
Novelty seeking	20.77 (5.34)	–	24.13 (6.83)	–	−2.10^b^	0.038	0.59^d^
Harm avoidance	19.83 (6.25)	–	20.75 (7.82)	–	−0.50^b^	0.618	0.14^d^
Self-transcendence	14.29 (6.56)	13.00	22.00 (7.58)	23.50	808.00^a^	**<0.001** *	**−0.38^c^**

BIS, Barratt Impulsiveness Scale. Bold denotes significant differences. *Denotes significance when controlling for multiple comparisons. ^a^Mann–Whitney *U* test; ^b^*t*-test; ^c^*r*; ^d^Cohen’s *d*.

## Discussion

The main objectives of this study were to examine gender differences in severity of PIU rates of psychiatric comorbidity, addictive behaviors, and personality characteristics among people seeking treatment for PIU. To our knowledge, the present study is the first to examine gender differences in PIU in a clinical sample of people seeking treatment for PIU. The results of our study found no significant gender differences in the demographic profiles of the sample. Our study consisted of a higher proportion of men compared to women, which is in line with previous studies among non-clinical samples that find a higher preponderance of PIU among men (25, 28, 29). Additionally, we found gender differences in the type of internet use that was problematic with men reporting greater problematic use of internet gaming compared to women. Taken together, the results of our study corroborate findings with non-clinical samples regarding the difference between men and women in problematic patterns of internet use.

Overall, the rates of psychiatric comorbidity were high in our sample. For instance, nearly half of participants met the criteria for major depressive disorder. The high rates of psychiatric comorbidity in our sample could be linked to the distress associated with PIU, which may exacerbate or result in comorbid psychopathology. It is also possible that psychiatric conditions have a bidirectional relationship with PIU. For instance, excessive internet use in individuals with PIU may be a coping mechanism to alleviate distress associated with co-occurring psychopathology (9) as is seen in excessive gambling (52) and compulsive sexual behavior (53). On the other hand, PIU may lead to mental health difficulties given the interpersonal and personal harms that are associated with PIU (4). Future research that investigates the temporal relationship between PIU and psychiatric comorbidities would be highly informative and aid in development prevention and treatment initiatives for PIU and associated psychopathologies.

In terms of gender differences in rates of psychiatric comorbidity, women were more likely to meet the criteria for major depressive disorder, suicidality, anxiety disorders, OCD, PTSD, and bulimia nervosa. In many cases, the rate was at least double in women compared to men. Women were also more likely to score higher on addictive behaviors including shopping and disordered eating, which is consistent with previous research on behavioral addictions (54). Taken together, these results may suggest that women with PIU present with greater clinical complexities and that the higher levels of distress and functional impairment, caused by excessive use of the internet or comorbid psychopathology, may be a particularly important factor in seeking treatment for PIU. Future research that examines the importance of addressing the comorbid psychopathology in treatment outcomes for PIU would be highly informative.

Finally, gender was associated with several personality characteristics in the current study. For example, women tended to score higher on impulsivity than men, particularity on motor impulsiveness. Though scores on impulsivity measures tend to be relatively similar across genders in the general population (55), some research suggests that women may score higher in populations with addictive and/or impulse control disorders (56), which is in line with the results of the present research. Further, women in the sample scored higher on self-transcendence than men, which tends to be stable between genders in cross-national research (57). Self-transcendence contains elements of spirituality and identification with the universe and has been associated with increased psychopathology including behavioral addictions such as gambling (58) and mood disorders (59). Thus, the higher self-transcendence in our sample of women seeking treatment for PIU may partially help to explain the higher rates of psychopathology in women.

The results from the present study not only contribute to the limited body of literature on PIU, but they also have important implications on its assessment and treatment. Although men are more likely to seek treatment for PIU, women who present with PIU are likely to present with greater clinical complexities. Indeed, women are more likely to report greater rates of psychopathology. As such, clinicians should be diligent about screening for a wide variety of disorders known to be linked to PIU, especially among women. Further, interventions should be individualized and aim to target co-occurring conditions where possible to optimize treatment outcomes. Specifically, transdiagnostic treatment options tend to yield more favorable outcomes (60, 61) and may be a good option to target both PIU and co-occurring psychological processes. Additionally, given the findings of greater psychiatric co-morbidities of women (and in the sample generally), an integrated treatment approach that targets both PIU and co-occurring mental health disorders may be of particular benefit. This approach would involve the treatment of PIU and co-occurring psychiatric disorders occurring simultaneously, and by the same team of practitioners (62). Previous studies have found integrated treatment options to lead to superior clinical outcomes and are more cost effective than non-integrated treatments (63).

### Limitations

A limitation of the present research is the relatively small sample size overall and in particular the small sample of women. Although correcting for multiple comparisons and the moderate effect sizes may provide some confidence in our findings, future research should include a more representative sample of women and other gender identities. Furthermore, although there were no significant differences in age between men and women, future studies that use an age matched design between men and women with PIU would be informative. A third limitation of the present research is that not all participants completed all measures of our interest. However, we conducted missing data analysis, which indicated that the data was completely missing at random. Though a strength of this study is its use of a clinical population to fill a gap in the existent literature, this could also be considered a limitation given that participants were all seeking treatment for their PIU and therefore are not likely representative of the general population. For example, the greater problem severity in the women in our sample may reflect that these women are more reluctant than men to seek addiction treatment and only do so when greatly impaired. They may, in contrast, be seeking treatment for comorbid conditions such as depression or anxiety. Fifth, we used a self-report measure of personality rather than an in-depth diagnostic tool. Lastly, given the exploratory nature of this study, the results presented here should considered preliminary evidence for future investigation of potential gender differences in PIU, particular among clinical samples. Specifically, future studies that examine more focused dimensions of psychopathology and personality would be beneficial.

## Conclusion

Our results ultimately suggest that there are notable gender differences in PIU severity and associated psychiatric and personality factors, with women presenting with greater PIU severity and increased psychiatric comorbidity. These findings may have implications on the clinical assessment and treatment of PIU in the future. Future research should seek to further investigate the nature and course of PIU in clinical populations to ensure timely prevention, identification, and treatment, especially amongst women.

## Data availability statement

The raw data supporting the conclusion of this article will be made available by the authors pending approval from the authors’ Research Ethics Board.

## Ethics statement

The studies involving human participants were reviewed and approved by the Faculty of Medicine, University of São Paulo’s Research Ethics Board and Ryerson’s Universities Research Ethics Board. The patients/participants provided their written informed consent to participate in this study.

## Author contributions

RM, HK, VO, and LS wrote the first draft of the manuscript. HK conducted the formal analyses. HT provided supervision and resources. LS and DH substantially edited the manuscript. All authors contributed to the conceptualization of the research and approved the submitted version.
